# Reproduction of Lactation

**Published:** 1852-07

**Authors:** 


*Reproduction of Lactation.* The *Am. Jour. of Med. Sci.* for Jan. contains a report of some cases read before the Rhode Island Medical Society, by Ariel Ballou, M. D., in which lactation was reproduced after an absence of from three to four months. Before seeing Dr. B.'s cases, the writer had occasion recently to recommend a similar course to a patient, in whom, in consequence of severe illness following confinement, the secretion of milk was suspended for several weeks. The result was entirely satisfactory.

The following is one of Dr. Ballou's cases :—

"The following case I report as having an important practical bearing on the treatment and disposal of a class of cases which occur in our community at the present day, to cure which, or otherwise dispose of satisfactorily to the physician, is often found difficult.

"Case III.—Mrs. O. H. H., aged about twenty-one years, of feeble constitution, and nervo-lymphatic temperament, was confined in July, 1847. Previous to her accouchement she was troubled with chronic aphtha, red canker, or with that condition of the system which is well known as "sore mouth attendant on pregnancy and lactation." Nothing unusual occurred at the time of delivery. No considerable loss of blood was sustained. As in similar cases, there was a remission of diarrhœa and sore mouth for a few days after accouchement, giving rise to a hope that, being relieved from the condition of pregnancy, she would recover the powers of digestion and the assimilation of nutriment, so as to enable the system to sustain the calls upon it consequent to lactation. But in the course of ten or twelve days after accouchement the sore mouth and diarrhœa returned with increased violence, producing great debility. The secretion of milk was copious; her pulse 120; the tongue flabby; there were frequent copious dejections of yellowish water, the face and extremities bloated, &c. Fearing the worst results from my patient, I advised the immediate removal of the child from the breasts of the mother to those of a wet-nurse, at the same time informing the parents that on the recovery of the mother, she could at pleasure reapply the child to the breast and have a full supply of milk, and be enabled to perform all the duties and functions of a mother for an indefinite period of time. The child was given in charge of a wet-nurse, the milk gradually disappeared, and the patient recovered under the use of tonic remedies and a generous diet. Between two and three months after this the mother called on me, having the appearance of restored health, and inquired if she might now take her child home with a hope of realizing my former assurances that she would be able to reproduce her milk. I assured her there was no doubt in relation to such a result, and her ability for the future to nurse her child. She took the child, applied it to the breasts, and in the course of two weeks had a good supply of milk.

"I met her some nine months after, when she informed me she was happy in the enjoyment of good health, and, to use her words, she 'had a good a breast of milk as if she had never dried it up.'"—*New Jersey Med. Reporter.*

